# GOAL ATTAINMENT SCALING IN CAPABLE: A COMPARISON OF CARE PARTNER AND OLDER ADULT PARTICIPATION

**DOI:** 10.1093/geroni/igad104.1882

**Published:** 2023-12-21

**Authors:** Kate Perepezko, Pamela Toto

**Affiliations:** University of Pittsburgh, Pittsburgh, Pennsylvania, United States; University of Pittsburgh, Pittsburgh, Pennsylvania, United States

## Abstract

Aging in place is an optimal outcome for our maturing population as health systems become increasingly overburdened. One program that facilitates aging in place is the Community Aging in Place, Advancing Better Living for Elders (CAPABLE) program. CAPABLE improves older adult performance in everyday activities and reduces inpatient and long-term service use. Although CAPABLE is designed as a person-centered program aiming to help older adults set and achieve goals, research demonstrates that care partners (i.e. family or friends providing unpaid support) are frequently involved in healthcare decisions and can reinforce intervention goals. This presentation will report findings from the trial stage of a Hybrid Type 1 Study of CAPABLE involving care partners for which recruitment is ongoing (N=100). We aim to describe how older adults and their care partners engage in goal setting using Goal Attainment Scaling, share the frequency of their goal attainment, and present the types of goals that they set. For example, older adult participant goals for our current sample focus on reduced fear when showering, improved mobility, and more efficient dressing. For our care partner participants, goals include finding respite and preventing older adult injury. All participants who have completed CAPABLE were able to achieve their goals by the end of the program. Findings from this study support care partner involvement in interventions to promote aging in place, aligning with the RAISE Act’s goal to engage family caregivers as key partners in the delivery of services and support.

